# Impact of COVID-19 infodemic on psychological wellbeing and vaccine hesitancy

**DOI:** 10.1186/s43168-021-00061-2

**Published:** 2021-03-05

**Authors:** Janmejaya Samal

**Affiliations:** Independent Public Health Researcher, Bhubaneswar, Odisha India

**Keywords:** Disinformation, Fake news, Internet media, Misinformation, Social media

## Abstract

**Background:**

With COVID-19 pandemic, the world has witnessed a scenario that is unique compared to any other such pandemic that the world has grappled with. This is primarily owing to the parallel infodemic that the population faced with disinformation and misinformation explosion in several platforms that an individual can access.

**Main body:**

The myriad of information that everyone in the world received acted as double-edged sword as some information helped individuals in allying the anxiety and stigma and motivated them for appropriate COVID-19 behavior; however, on the other hand, the same acted opposite and created a whole lot of negative problems in the community. The misinformation regarding the disease is not only limited to what has happened so far in the realm of prevention and control rather the same is also plaguing the efforts towards effective vaccine uptake.

**Conclusion:**

With the technological and media advancement, it is getting difficult to ward off every misinformation that is getting received at individual end which is obviously detrimental in the efforts toward effective vaccine acceptance; however, measures need to be taken at appropriate level to curb this menace of infodemic to relax the world from the clutch of this pandemic. This article looks at the impact of the misinformation and disinformation on psychological wellbeing and vaccine acceptance and suggests remedial measures.

## Background

The Wuhan city of Hubei Province, China, witnessed several cases of pneumonia with unknown etiology during the last week of December 2019 which started spreading to other parts of China and the world after a couple of days [1]. It was observed that these cases reported the health facility with fever and cough and have a history of contact to Huanan seafood market [2]. A novel coronavirus in the throat swab sample of one patient was identified by the Chinese Centre for Disease Control and Prevention (CDC) on 7 January 2020, and subsequently, the World Health Organization (WHO) named the virus as 2019-nCoV [3]. The World Health Organization declared the outbreak as public health emergency of international concern (PHEIC) in January 2020 [4]. The International Committee on Taxonomy of Viruses renamed the virus as severe acute respiratory syndrome coronavirus 2 (SARS-CoV-2) on 11 February 2020 [5], and the disease was named as coronavirus disease 2019 (COVID-19) [6]. By now, many achievements have been accrued especially in the front of virus identification, understanding clinical manifestations, and diagnosis of the disease; however, no effective treatment has been found out till date [7–9]. Furthermore, finding an appropriate vaccine can only be the solution to protect the entire population of the world.

## Main text

### What is infodemic

After the declaration of the outbreak as a public health emergency of international concern by the WHO, soon after, the WHO declared that the new coronavirus pandemic is accompanied by an “infodemic” of misinformation [10]. It has further been described as a “second disease” accompanying the pandemic by the WHO. Infodemic is the overabundance of information both correct and false that puts people in confusion to accept or reject when they need it [11]. The misinformation could be of two different kinds: “disinformation” is meant for circulation with malicious intentions and the “misinformation” is meant to spread lies with or without bad intentions. Irrespective of the category, both are harmful, and the matter of concern here is about pandemic and human health [12].

### Impact of “infodemic” on psychological wellbeing

While fighting infodemic was mulled over based on factuality, however, the same is a much broader problem, as malicious content not only includes fake news, rumors, and conspiracy theory but also the promotion of fake treatment modalities, panic, racism, xenophobia, and mistrust, among others [13]. Researches have shown that the impact has been substantial on mental health and wellbeing. One of the publications that systematically analyzed 225 misinformation pieces from January to the end of March 2020 from a corpus of English language fact checks gathered by the First Draft News, emphasizing on the content rated false and misleading, found that 88% of the false information appeared in social media platforms and, in addition, 9% in TV, 8% in news outlets, and 7% appeared in other websites [14]. At the beginning of the pandemic, the entire world was under the clutch of misinformation and the social and internet media exploded like never before on any such public health issue, and nobody could really understand what is happening in the realm of health education and whether people are getting right information from right sources or wrong information from “right-look-like” sources that created a whole lot of confusion. Moreover, people are vulnerable to misinformation and biased information owing to their own belief system, culture, and the level of education and are less aware about the authenticity of the sources of information. One of the clinical professors of pharmacy has published a dose of inspiration in which she says “COVID-19 era of misinformation—when your family does not trust you, will your patients?” which clearly reflects the impact of infodemic on the COVID-19 pandemic [15]. This is one such example where a healthcare professional has to witness distrust in her own family and would have happened to many such health professionals in their families where a family member is ready trust and get guided by a piece of information or may be a piece of misinformation which may not necessarily be correct because these mis/information were presented in such a fashion that people tend believe them more than that of a right information available at their home/family itself. The current trend shows that the actual messages from the mass media gets further amplified in social media and served for larger consumption and have become immensely popular [12]. It has been found out that much of the public understanding about health issues and policies do not really come from their direct experience but rather from what they read and understand through the media [16]. Research has shown that a substantial portion of the message that gets circulated in social media does not come from reliable sources. Hence, problematic social media usage led to more misunderstanding about COVID-19.

One of the studies that investigated the mediated effects of fear of COVID-19 and misunderstanding of COVID-19 associated with problematic social media usage found that problematic social media usage is significantly associated with psychological distress and insomnia among a sample of 1078, both directly and indirectly [17]. It is further substantiated by the evidence that when people receive a greater amount of misinformation and misconceptions of COVID-19 through social media usage, their uneasiness is increased which subsequently gets converted in to psychological distress [18]. Another study on the impact of social media on public worry among the Taiwanese population through an online survey revealed different sources of information and current and past worries associated with misinformation. The sources of information in percentage were internet media (80.52), traditional media (52.62), co-workers (23.57), family members (24.36), friends (21.08), academic courses (21.18), and medical staffs (19.03). The study revealed that the information associated with internet media, traditional media, and friends is associated with highest level of current worry [19]. A study that conducted YouTube video analysis found that of the 69 videos analyzed, 27.5% of the videos contained non-factual information and had 62,042,609 views, meaning spread of public worries and panic to approximately these many people [20]. An online survey using convenient sampling technique in the USA found that around two thirds of the participants were worried about getting infected and the source of medical help should they be needed. It was further found that 95% of the participants changed their behavior with the fear of getting infected and resorted to COVID appropriate behavior [21]. A study in Iraq found that fear and panic about COVID-19 were higher among the social media users, and it has negatively impacted the mental health status of approximately half of the social media users in the country [22]. Interestingly, it was found in China that the impact of misinformation on mental health is dose dependent. The longer the duration of exposure to social media, the more is the reported anxiety and depression [23].

### Impact of infodemic on COVID-19 vaccine behavior

Akin to the impact of infodemic on psychological wellbeing, the infodemic can also have a detrimental effect on the acceptance of vaccine across several individuals causing widespread vaccine hesitancy. Vaccine hesitancy refers to delayed acceptance or refusal of the vaccine despite the availability of the same [24]. The 13th Meeting of the Immunization Practices Advisory Committee of WHO held on 11 to 13 June 2019 reminded that increased vaccine hesitancy coupled with the misinformation spread by the anti-vaccination group is a serious threat in controlling vaccine-preventable disease. Hence, the WHO recently listed vaccine hesitancy as 10 threats to global health [25]. Vaccine hesitancy has been a global trend these days and has been reported by approximately 90% of the countries in the world [26]. Vaccine hesitancy is a complex phenomenon and is context specific varying across time, place, and vaccines. The model of vaccine hesitancy consists of three “Cs”—confidence, complacency, and convenience. It is most widely understood that a high level of hesitancy leads to low vaccine demand; however, on the contrary, low hesitancy does not necessarily lead to high demands of the vaccine [27]. Outbreaks of several vaccine-preventable diseases are associated with vaccine hesitancy such as Haemophilus influenza type B (HiB), varicella, pneumococcus, and pertussis [28, 29]. This is primarily a people-made crisis despite the unequivocal fact that vaccination remains the most significant public health interventions of our time [30, 31]. Given the context, the misinformation is also going to impact vehemently towards the efforts of COVID-19 vaccination [32].

Several surveys conducted during the recent past shows a wide range of COVID-19 vaccine hesitancy among different populations in the world. One of the recent surveys conducted by an American public health school found that 12% of the American residents would not take the vaccine and 82% of them are concerned about the safety of the vaccine [33]. Another cross-sectional study conducted among university students based on a convenient sample in Italy revealed that one of the 10 students did not show positive intention to take vaccine, showing vaccine hesitancy among 13.9% of the students [34]. An online survey in the UK (*N* = 1088) and Turkey (*N* = 3936) revealed that 14% and 31% of the study participants in UK and Turkey, respectively, were unsure of accepting a potential COVID-19 vaccine and 3% of the study participants straight away rejected the acceptance of the vaccine [35]. A demographically representative sample of 5009 American adults were surveyed between May and June 2020 and found that 31.1% of the residents were not interested to take the vaccine. Among the refusal group, the likelihood was more among Blacks, women, and conservatives [36]. A two-phase representative sample, phase 1 (*n* = 968) and phase 2 (1004), were surveyed to assess the acceptability of the vaccine among Italian population. The study revealed that 41% of the participants in phase 2 reported of vaccine hesitancy. The study revealed a greater level of mistrust on bio-medical research among the study participants [37]. Two political leaders in Pakistan raised concerns over the vaccine and stated that the vaccine is a grand illusion and a conspiracy against the Muslim countries. Such narrative could undermine the efforts of COVID vaccination in the country [38]. A global survey conducted in 19 countries that represent 55% of the world’s population on a random sample among a population of 13,426 assessed the acceptability of a potential COVID-19 vaccine. The highest positive response was obtained from the sample of Chinese population in which 88.6% (*n* = 712) responded positive for the vaccination if provided with a proven, effective, and safe vaccine. Respondents from Poland gave the highest percentage of negative response (27.3%, *n* = 666) [39]. A survey conducted in early November 2020 by the World Economic forum among 18526 participants of 15 countries revealed that only 73% of people are willing to take the vaccine showing a four-point fall since August [40].

Similarly, a survey in India by citizen survey platform Local Circle revealed that 69% of the respondents see no urgent need of getting vaccinated. The survey also revealed that limited information about side effects, efficacy level, and belief of higher level of immunity are the major factors of hesitancy [41]. India is consistently grappling with the issue of vaccine hesitancy as in the year 2000 Muslim population in Uttarpradesh had the misconception of oral polio causing infertility and is ineffective causing 5 times low uptake of oral polio vaccine. In 2016, a survey found a low uptake of diphtheria vaccine among the Muslims of Kerala and low uptake of measles and rubella in 2017 in Tamilnadu and Karnataka. The major factors were fear, misinformation spread on social media, and lack of awareness [40].

### Managing infodemic

The COVID-19 infodemic could be severely detrimental in the efforts towards controlling, preventing, and managing the disease and the same is also equally applicable to vaccine acceptance. Thus, it becomes imperative that the infodemic is managed appropriately. This was rightly said by the Director General of WHO Dr. Tedros Adhanom Ghebreyesus “we are not just fighting an epidemic; we are fighting an infodemic” in a Munich Security Conference on 15 February 2020 [41]. The following few points can help manage the infodemic appropriately:

- *Responsible health journalism*: the media plays an important role in spreading both right and wrong information directly and indirectly; hence, it becomes imperative to adhere to responsible journalism. Qualified and professionally trained full-time reporters should be involved in making health stories. The media house should take the pain of checking the facts and demystify fake news, disinformation, and misinformation. There should be convergence between media house and health experts to improve the quality of health news. Above all, the media should take the responsibility of empowering and educating the masses through appropriate and accurate information [42].
- *Leaders in responsible portfolios should mark their words*: politicians in responsible positions should mark their words while making a public statement as their statements are taken seriously and followed by everybody in the community. They particularly should refrain from downplaying the seriousness of such pandemic without proper technical advisory. During the COVID pandemic, many of the statesmen’s statements came in to surface which downplayed the seriousness of the disease putting the citizens’ life at stake. Ironically, many of them ridiculed the significance of this pandemic and were contradicting to what public health organizations and scientists said. These statements in traditional and social media gets converted in to misinformation and disinformation and spread like wild fire [43].
- *Responsible information seeking and sharing*: needless to say, the hunger to consume information about a disease during pandemic is enormous. People would be seeking as much information as they could to gratify their information appetite and would also share the same with others as well. However, while doing so, it is the responsibility of the information seeker to absorb information from credible and appropriate scientific sources and do share the same. People should also refrain from engaging themselves in participating in discussion forums where misinformation or disinformation is being shared [44].
- *Responsible information sharing by appropriate authorities*: public health specialists, medical doctors, and scientists should take the responsibility and lead in sharing authentic, useful, and transparent information to public through appropriate fora such as interviews, podcasts, op-eds, blogs, and social media [45].
- *Linking of search engines to appropriate scientific sites*: major and prominent search engines such as “Google” and “Yahoo” should direct to appropriate scientific sites with the input of keywords at the top of the webpage. This would help in preventing people from browsing misleading sites and getting inaccurate information. This is true with the findings of one of the studies where discrepancy in the usage of masks and hand washing is found as a result of browsing different sites [46].
- *Empathetic communication*: pandemic situations like COVID-19 pandemic requires empathetic communication as the same can allay the anxiety and infuse compliant behavior towards positive help and prevention seeking behavior. This is important as people were found to be low engaged with government agencies and found more attracted towards posts that were more personal, empathetic, and expressed worry and concerns about the pandemic [47].
- *Communication for specific groups*: specific communication strategies should be framed towards minority groups, classes, races, and ethnicities. Role models from special groups should be promoted to create awareness so that people from same community feel relevant and would receive the awareness properly [48].
- *Educational material directed towards health education*: educational materials should be designed to educate people as during the pandemic people do have high appetite towards consuming information from many different sources. Thus, appropriate health education should be made through the traditional media to educate the common mass. It is believed that message from traditional media will eventually flow to the social media and will spread rapidly and if the information is accurate it will help creating awareness [11].

## Conclusion

The infodemic is as serious as the pandemics and can spread at higher speed than that of the pandemic. Hence, it becomes significant and imperative that the infodemic is encountered appropriately and timely in order to promote health and wellbeing and aid in the prevention, vaccination, and management of pandemic of COVID-19. Given the context, the media, both traditional and social, should play a robust role in creating awareness, promoting healthy habits, making people exposed to accurate information, and improving psychosocial wellbeing. Above all, the government should play an active role in regulatory roles, bringing polices that would govern the media in mitigating the menace of the infodemic.

## Data Availability

Not applicable.

## Abbreviations

CDC: Centre for Disease Control and Prevention
COVID-19: Coronavirus disease 2019
HiB: Haemophilus influenza type B
PHEIC: Public Health Emergency of International Concern
SARS-CoV-2: Severe acute respiratory syndrome coronavirus 2
UK: United Kingdom
USA: United States of America
WHO: World Health Organization
